# Acknowledgment to reviewers—January 2025 to October 2025

**DOI:** 10.1093/fsr/owaf048

**Published:** 2026-01-21

The Editors of the *Forensic Sciences Research* (*FSR*) wish to thank the following people, and any other reviewer whose name has been inadvertently omitted, for giving their time and expertise to review papers and facilitate the smooth running of *FSR* between January 2025 and October 2025.

Ghadeer Abdelaal, *Egypt*

Maxwell Abedi, *Ghana*

Halide Açıkgöz, *Türkiye*

Agostinho Almeida, *Portugal*

Andreas Alt, *Germany*

Anna Barbaro, *Italy*

Ilenia Bianchi, *Italy*

Patrícia José Anastácio Jardim, *Portugal*

Bill Buck, *USA*

Francesco Paolo Busardò, *Italy*

Melina Calmon, *Brazil*

Andrea Luigi Cardini, *Italy*

Hugo F. V. Cardoso, *Canada*

María Josefina Castagnola, *USA*

Man Chen, *China*

Shitao Chen, *China*

Hao Chen, *USA*

Liping Chen, *China*

Peng Chen, *China*

Xiaohong Chen, *China*

Alberto Chighine, *Italy*

Xavier A. Conlan, *Australia*

Maria Begoña Criado, *Portugal*

Paulo Dario, *Portugal*

Jan De Kinder, *Belgium*

José Orlando de Almeida Silva, *Brazil*

Júlio de Carvalho Ponce, *Brazil*

Ward De Spiegelaere, *Belgium*

Wanyu Deng, *Singapore*

Giancarlo Di Vella, *Italy*

Matteo Donghi, *Italy*

Isabel Draper, *Spain*

Sarah Ellingham, *Switzerland*

Metin I. Eren, *USA*

Belén Estébanez, *Spain*

Kevin J. Farrugia, *UK*

Rufino Felizardo, *France*

Roy Fenoff, *USA*

Claire Ferguson, *Australia*

Santo Davide Ferrara, *Italy*

Alex Stewart Forrest, *Australia*

Lorenzo Franceschetti, *Italy*

Charlie Frowd, *UK*

Rodrigo Garrido, *Brazil*

Benjamin Gearey, *UK*

Denise Gemmellaro, *USA*

Janice Glime, *USA*

Justin Z. Goldstein, *USA*

Yucheng Guo, *China*

Mukaddes Gürler, *Türkiye*

Yinan He, *China*

Chong Chin Heo, *Malaysia*

Kevin C. Honeychurch, *UK*

Kenneth K. Kidd, *USA*

Ikram Kort, *Tunisia*

Cheng-Lung Lee, *Taiwan*, *China*

Shufeng Li, *China*

Liqing Li, *China*

Haozhe Li, *China*

Xin Liao, *China*

Rafael Linden, *Brazil*

Xiling Liu, *China*

Luoxi Liu, *China*

Bing Liu, *China*

Aurelio Luna, *Spain*

Klaus Lunau, *Germany*

Yehui Lv, *China*

Filomena Melchionda, *Italy*

Florian Menzel, *Germany*

Xeniya E. Mkhitaryan, *Kazakhstan*

Anabela Pereira Neves, *Portugal*

Emilio Nuzzolese, *Italy*

Zuzana Obertová, *Australia*

Tsukasa Ohuchi, *Japan*

Antonel Olckers, *South Africa*

Tommaso Pacini, *Italy*

Jonathan Parrott, *USA*

Guido Pelletti, *Italy*

Maria Alejandra Perotti, *UK*

Anita Pettersson-Strömbäck, *Sweden*

Brian Schou Rasmussen, *Denmark*

Hugo Rodríguez Almada, *Uruguay*

Koichi Sakurada, *Japan*

Rick Schulting, *UK*

Kerem Sehlikoğlu, *Türkiye*

Ayoub Simou, *Morocco*

B. Holly Smith, *USA*

Ruiyang Tao, *China*

Benito Soto-Blanco, *Brazil*

Aaron Tarone, *USA*

Diana Toneva, *Bulgaria*

Pedro A. Torres-Carrasquillo, *USA*

Alice Towler, *Australia*

Leonid N. Valentovich, *Belarus*

Maria Isabel de Oliveira e Britto Villalobos, *Brazil*

Richard L. Wall, *UK*

Zheng Wang, *China*

Yu Wang, *China*

Le Wang, *China*

Hong Wang, *China*

Caroline Wilkinson, *UK*

Carl Johan Wingren, *Denmark*

Hui Yan, *China*

Yifeng Yao, *China*

Lagabaiyila Zha, *China*

Junyu Zhang, *China*

